# Major air pollution and climate policies in NYC and trends in NYC air quality 1998–2021

**DOI:** 10.3389/fpubh.2024.1474534

**Published:** 2024-10-16

**Authors:** Kathleen Lau, Jia Guo, Yuqi Miao, Zev Ross, Kylie W. Riley, Shuang Wang, Julie Herbstman, Frederica Perera

**Affiliations:** ^1^Columbia Center for Children’s Environmental Health, Columbia University, New York, NY, United States; ^2^Department of Biostatistics, Mailman School of Public Health, Columbia University, New York, NY, United States; ^3^ZevRoss Spatial Analysis, Ithaca, NY, United States

**Keywords:** policy, air pollution, child health, climate change, justice

## Abstract

**Introduction:**

Air pollution poses serious health risks to humans, with particular harm to children.

**Objectives:**

To address the gap in understanding the efficacy of policies to reduce exposure to air pollution, we sought to assess the temporal relationship between the enactment of major air pollution and climate policies in NYC and trends in air quality during the period 1998–2021. We used previously available data from citywide monitoring and new data from the Columbia Center for Children’s Environmental Health (CCCEH) longitudinal cohort studies of mothers and children living in communities in Northern Manhattan and the South Bronx.

**Methods:**

We utilized publicly available citywide air monitoring data for particulate matter (PM_2.5_) and nitrogen dioxide (NO_2_) from 2009 to 2021 from the New York City Community Air Survey (NYCCAS) database and CCCEH cohort data on residential exposure to PM_2.5_ and NO_2_ and personal exposure to polycyclic aromatic hydrocarbons (PAH) during pregnancies occurring from 1998–2016 and 1998–2021, respectively. We compared annual and overall reductions in PM_2.5_ and NO_2_ citywide and reductions in PAH concentrations in the cohort studies.

**Results:**

As previously reported, annual average concentrations of pollutants in NYC dropped significantly over time. Between 1998 and 2021, PM_2.5_ and NO_2_ concentrations were reduced citywide by 37 and 31%, respectively. In our CCCEH cohorts, between 1998 and 2016, the annual average PM_2.5_ and NO_2_ concentrations also decreased significantly by 51 and 48%, respectively. Between 1998 and 2020, PAH concentrations decreased significantly by 66%.

**Discussion/conclusion:**

While it is not possible to link improved air quality to a single policy, our analysis provides evidence of a cumulative beneficial effect of clean air and climate policies enacted between 1998 and 2021 both city-wide and in our cohorts residing in communities that have been disproportionately affected by air pollution. There are important implications for health benefits, particularly for children, who are known to be especially vulnerable to these exposures. The results support further environmental and social policy changes to prevent the serious health impacts of air pollution from fossil fuel emissions.

## Introduction

To assess the impact of policies enacted in New York City aimed at improving air quality and curbing climate change, we have evaluated trends in levels of particulate matter 2.5 microns or less (PM_2.5_), nitrogen dioxide (NO_2_), and polycyclic aromatic hydrocarbons (PAH) over the relevant time period. Air pollution, notably PM_2.5_ and NO_2_, largely from fossil fuel combustion, is detrimental to human health and has been associated with acute and chronic negative health effects including adverse birth outcomes, neurodevelopmental and neurodegenerative disease, infant death, and asthma and other respiratory disease in adults and children (1–5). Air pollution is also a risk factor for respiratory infections, bronchitis, and impaired lung growth and function (6). These health effects may persist or worsen in adulthood. The fetus and child are especially vulnerable to air pollution due to their rapid and complex developmental programming that can be readily disrupted by exposure to toxicants, immaturity of biologic defense and immune systems, and a host of other biologic and behavioral factors (1–4, 7–9). Compared to adults, infants and children breathe more air relative to their body weight, and have smaller airways that are more readily constricted by particulates and allergens. In New York City and other urban areas of the US, there is disproportionate exposure to air pollution in communities of color and low-income communities (10, 11). Disparities also exist in rates of the outcomes linked to air pollution exposure (12, 13).

The US and other countries have enacted policies designed to lower levels of air pollution and address the growing threat of climate change; these have had substantial health and economic benefits (14). Examples include the 1990 Clean Air Act Amendments in the US that led to significant reductions in levels of fine, respirable particulate matter (PM_2.5_) and nitrogen dioxide (NO_2_), with major associated health benefits including reductions in premature mortality, acute myocardial infarction, asthma exacerbations, and hospital admissions for respiratory conditions (15, 16). The Clean Air Act has also reduced racial/ethnic disparities in air pollution exposure (17). Other countries have seen similar benefits for policies aimed at reducing air pollution. In Beijing, China, a 35% reduction in fine particles from 2013 to 2017 has been attributed to clean air policies (18). A study in Krakow, Poland found that policies to reduce coal burning between 2010 and 2019 were associated with a 39% reduction in annual average PM_2.5_ (19). In the past, assessments of the benefits of clean air policies generally focused on adult mortality. Increasingly, however, children’s health outcomes are being included (20–22).

A previous review of legislation in NYC catalogued laws enacted between 1998 and 2017, systematically sorting them into categories of selected risk factors for cardiovascular disease including air pollution (23). We extended this review to 2021, focusing on major legislation addressing emissions of air pollutants including PM_2.5_, NO_2_, and polycyclic aromatic hydrocarbons (PAHs), a class of combustion- and traffic-related hazardous air pollutants (24). Unlike PM_2.5_ and NO_2_, PAH are not regulated as criteria pollutants under the Clean Air Act. Among the key policies identified are the Clean Fuel Bus Program, Clean Air Taxi legislation, and Clean Heat Program, which we and others have previously assessed in terms of air quality improvement (25). The NYC Clean Air Taxi legislation is composed of several laws intended to regulate emissions from the NYC medallion taxi fleet, also referred to as “yellow cabs,” by mandating and incentivizing the integration of hybrid vehicles, compressed natural gas vehicles, and other low-emission vehicles into the taxicab fleet, as described in Appendix Table 1 (26). We estimated that the Clean Air Taxi legislation reduced taxi fleet emissions of NO_2_ by 82%, and total particulate matter (TSP) by 49% between 2009 and 2015 (26). The Clean Bus Program, enacted to lower traffic emissions from the New York City bus fleet, also substantially reduced levels of nitrogen oxide (NO), NO_2_, and black carbon throughout the city between 2009 and 2014 (27). The Clean Heat Program, phasing out dirty residual heating oil in buildings, resulted in reduced ambient levels of sulfur dioxide (SO_2_), PM_2.5_, and NO_2_ (25, 28, 29). Prior analyses by others have found improvement in NYC air quality over time and suggested links to various clean air policies, but concluded that more action is needed to prevent health effects (25, 30–34).

Here, for the first time, we compare the trends in air quality for PM_2.5_, NO_2_, and PAH between 1998 and 2021 at the level of the city and at the personal level among our cohorts of pregnant women residing in Northern Manhattan and the South Bronx, communities overburdened by fossil fuel pollution.

## Methods

Like other large cities in the US and elsewhere, NYC has been a laboratory for policies addressing major environmental problems. Since 1998, the Columbia Center for Children’s Environmental Health has been carrying out prospective cohort studies of pregnant mothers and their children residing in the underserved communities of Northern Manhattan and the South Bronx in NYC. Extensive data have been gathered on exposure to air pollution and associated health effects, placing us in a position to assess the efficacy of policies in reducing these exposures.

We reviewed policies identified by Rhodes-Bratton et al. (23) aimed at mitigation of air pollution or climate change that were enacted in NYC over the period 1998–2017 and identified additional policies enacted through 2021. We considered those policies that had been reported in the peer-reviewed literature to have resulted in significant mitigation of air pollution in NYC to be “major policies” (see Appendix Table 1). Because analyses of their impact on air quality in NYC were not available for statewide legislation such as the New York Vehicle Inspection Program (NYVIP), regional legislation such as the Regional Greenhouse Gas Initiative (RGGI), or national legislation such as the Clean Air Act amendments and fuel efficiency standards for vehicles, these were not included, although they undoubtedly affected air quality in New York City.

## Citywide air monitoring

Citywide data on PM_2.5_ and NO_2_ from 2009 to 2021 were sourced from the database of the New York City Community Air Survey (NYCCAS), operated by the New York City Department of Health and Mental Hygiene Bureau of Environmental Surveillance and Policy^1^ (25). During this period, NYCCAS monitored airborne PM_2.5_, nitrogen oxides (NO_X_), SO_2_, ozone (O_3_), and black carbon (BC) during each season of the year at 60–150 locations throughout NYC. The number of sites changed over the years as the New York City Department of Health and Mental Hygiene learned about sources of and variation in air quality in NYC (25). Data were collected hourly over 2-week intervals at each of the distributed locations once per season, as well as at five reference sites year-round for the purpose of temporally adjusting distributed site data.

The downward trends in NYC air pollution have been previously reported (25). We referenced demographic data from the 2018 American Community Survey to explore whether the benefits were distributed in an equitable manner across NYC counties with differing percentages of people of color and people of low income (35).

### Cohort-based modeling and monitoring of air pollution

Three prospective cohorts are included in this analysis. The participants were drawn from communities in Northern Manhattan and the South Bronx, which are low-income communities directly served by the Mailman School of Public Health and CCCEH. The goal of the prospective cohort studies was to examine the relationship between exposure to air pollution and other toxicants on the health and development of children beginning *in utero* and extending through adolescence.

#### Mothers and newborns cohort

The Mothers and Newborns (MN) cohort includes 727 self-identified African American and Hispanic participants aged 18–35 years recruited between 1998 and 2006. Participants who initiated prenatal care after the 20th week of pregnancy, had a multiple pregnancy, smoked or used any tobacco product or illicit drugs, had diabetes or hypertension, were HIV positive, or had not resided in New York City for at least a year were excluded from the study.

#### Sibling/Hermanos cohort

The Sibling/Hermanos cohort began in 2008, when participants enrolled in the MN cohort who became pregnant with a subsequent child were invited to enroll. The Sibling/Hermanos cohort includes 129 siblings of the children in the MN cohort.

#### Fair start cohort

The Fair Start cohort began recruiting mother–child pairs in 2015. All participants are age 18+, speak either English or Spanish, and 90% self-identify on survey as Hispanic (36). The first 126 Fair Start participants were included in this analysis, as personal backpack monitoring was discontinued in March 2020 because of the COVID-19 pandemic.

### Modeling of exposure based on residential addresses for CCCEH cohorts

Within Mothers and Newborns and Sibling/Hermanos cohorts, exposure to PM_2.5_ and NO_2_ at participants’ residences was estimated using validated spatio-temporal models described previously in Ananth et al. (36) and Huang et al. (37) The models were developed using two sources of data: the New York City Community Air Survey (NYCCAS) data provided directly from New York City Department of Health and Mental Hygiene staff and regulatory data from the Environmental Protection Agency’s Air Quality System (AQS; https://aqsdr1.epa.gov/aqsweb/aqstmp/airdata/download_files.html) (38). At each NYCCAS site, 2-week integrated average samples were collected for each of the four seasons. The 2-week averages were converted to daily measurements by repeating the 2-week value for each day. To capture daily air quality variation in the models, daily data were included from the Department of Environmental Conservation’s regulatory monitors, which collect data on an every-day or every-third-day schedule. Depending on the year, there were between 16 and 23 regulatory monitors for PM_2.5_, and three to four monitors for NO_2_.

We computed the amount/density of candidate spatial predictors such as traffic and land use-related variables (derived from the New York City Department of City Planning’s Taxlot database) within 4 different buffer areas around each monitor site: 100, 300, 500, and 1,000 m (39). We also included several temporal predictors and computed daily city-wide averages. These include temperature, relative humidity, and wind speed. In addition, we computed the mixing depth and air-mass trajectory for each day.

The model was validated as described in Ananth et al. (40). The model was validated as described in Ananth et al. (40). Briefly, based on a 10-fold cross validation with an 80%/20% split we identified gradient-boosting machine models as the best fitting models for both PM_2.5_ and NO_2_ with cross-validation r2 values of 79% (74% when applied to the testing dataset) and 78% (69% when applied to the testing dataset) respectively.

The level of exposure for each day of the pregnancy was estimated for the two pollutants using model predictions. All exposure assignments were at a spatial resolution of 25 × 25 m^2^. Specific details on the exposure modeling and assignment have been previously published (40). We did not have data on time spent in and out of the home and daily activity patterns significant.

### Measurement of PAH exposure using personal backpack monitors

Within all three cohorts, we conducted personal monitoring of PAH using backpack monitors, as previously described. Personal air monitors were worn in backpacks by women in these cohorts in their third trimester of pregnancy during daytime hours for 2 consecutive days and placed near their beds at night (41). The air monitors were comprised of a continuously-operating pump, a precleaned quartz microfiber filter, and a precleaned polyurethane foam cartridge backup. Vapor and particles ≤2.5 μg in diameter were continuously collected by the pump, and analyzed at Southwest Research Institute (SwRI) for eight PAHs: benz[a]anthracene, chrysene, benzo[b]fluroanthene, benzo[k]fluroanthene, B[a]P, indeno[1,2,3-cd]pyrene, dibenz[a,h]anthracene and benzo[g,h,i]perylene. No monitoring data are available for the years 2007 and 2012–2015 because the last MN cohort birth occurred in 2006, the first Sibling pregnancies occurred in 2008, and the first Fair Start birth occurred in 2015.

We previously validated 48-h personal monitoring as an indicator of longer-term, integrated exposure. In a subset of the MN cohort (*n* = 84), indoor air was monitored over 6 weeks during the participants’ third trimesters concurrent with the personal air monitoring. The prenatal personal air concentrations were significantly correlated with indoor levels of PAH (sum of the 8 PAH; r = 0.58, *p*-value < 0.001) (42).

In analyses of trends in PAH exposure by season, October 16 to April 15 was designated as the heating season, while April 16 to October 15 was considered the non-heating season (43). The heating season is defined as the time period in NYC when building owners legally must maintain an indoor temperature of at least 68°F between 6 AM and 10 PM when the outdoor temperature is below 55°F, and at least 62°F regardless of the temperature outside between 10 pm and 6 am (44).

### Statistical analysis

#### Citywide trends in PM_2.5_ and NO_2_

Citywide trends were analyzed using simple linear regression models, with the PM_2.5_ or NO_2_ yearly averaged measures for the 13 years from 2009 to 2021 as the dependent variable and year index i = 1,…,13 as the independent time variable. Using these regression models, we estimated the yearly change of PM_2.5_ or NO_2_ over the time period 2009–2021 using city-wide data.

#### Trend in air quality at cohort participant residences

To assess the trend in air quality using data available from two of the three CCCEH cohorts (844 participants), using participants’ residence, we calculated participant-level PM_2.5_ and NO_2_ exposure measures averaged daily measurements over the entire pregnancy. Simple linear regression models (Equation 1) using participant level data were used to estimate the change in air quality over the years since the first date of birth (March 16, 1998). To construct the time index for each participant (i.e., number of years since the first birth in the MN cohort), we calculated the number of days between each date of birth and March 16, 1998 and divided by 365. For example, the participant with the earliest date of birth would have 0 days since the first date of birth. If a participant’s date of birth was March 17, 1998, this participant would have 1 day since the earliest date of birth, translating to 1/365 years since the earliest date of birth. Using these regression models, we estimated the yearly change of PM_2.5_ or NO_2_ over the time 1998–2016 using residential-level data.


(1) $$E\left(\text{exposure}|\text{time}\right)=\beta_{0}+\beta_{1}\text{time}$$

Where $E\left(\text{exposure}|\text{time}\right)$ denotes the expectation of exposure level of a participant given the time index, i.e., the number of years between the participant’s DOB and the earliest DOB in the MN cohort. ${ß}_{0}$ denotes the expected exposure level for the participants with the earliest DOB, and ${ß}_{1}$ is the expected change of the exposure level for participants with DOB 1 year later than the earliest DOB in the MN cohort.

To assess differences in the trend over time in air pollution levels between the heating and non-heating seasons, as defined above, we conducted stratified regression modeling. To assign a heating season indicator for each pregnant person, we calculated the number of gestational days that fell in the heating season (October 15 to April 15 of the following year). If this number exceeded 50% of days in the pregnancy, the person was assigned to the heating season.

#### Trend in PAH by personal monitoring in CCCEH cohorts

We used the simple linear regression models in Equation 1 to estimate the yearly change in log-transformed PAH using participant-level data using personal air monitors. To construct the time index for each participant (i.e., number of years since the first date of monitoring in the MN cohort), we calculated the number of days between each participant’s date of monitoring and first date of monitoring, February, 25, 1998 and divided by 365. We also stratified data by whether monitoring occurred in the heating or non-heating season.

## Results

### Citywide trends

**Figure 1:**
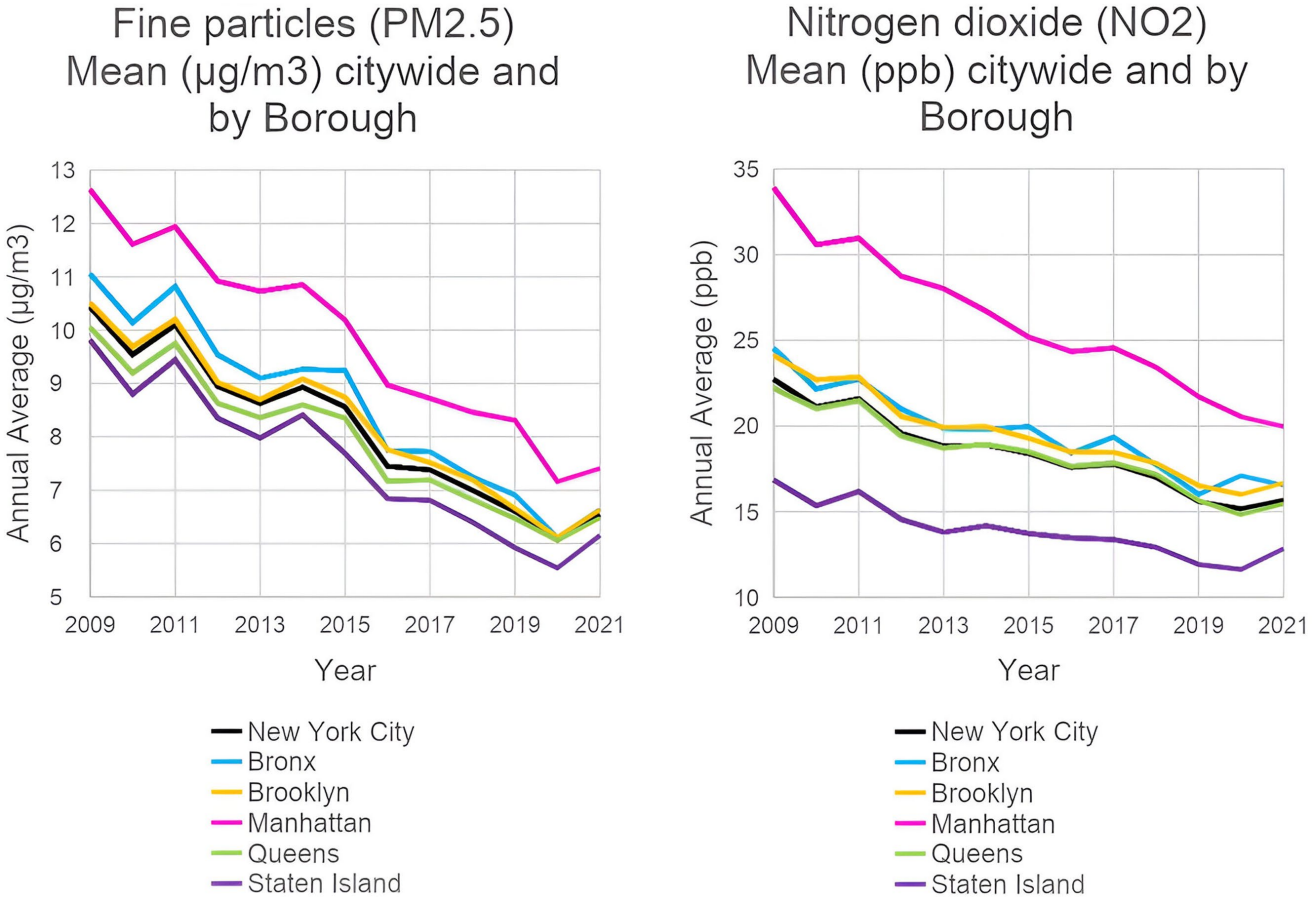
Ambient PM_2.5_ and NO_2_ levels in New York City from 2009 to 2021: city-wide and by borough. (EH Data Portal, NYC Department of Health and Mental Hygiene, 2022).

Comparing 2009 to 2021, there was a reduction of 3.89 μg/m^3^ (37.3%) in annual average PM_2.5_ concentrations, and a reduction of 7.04 ppb (31.0%) in annual average NO_2_ concentrations citywide. Using the linear regression model, there was a significant decreasing trend over time for both PM_2.5_ and NO_2_ (both with *p*-value = <0.0001; Table 1; Figure 1). We estimated a decrease of 0.35 μg/m^3^ in PM_2.5_ and a decrease of 0.59 ppb in NO_2_ concentration each year over all boroughs of NYC. We note that these decreases between 2009 and 2021 are slightly lower than those reported by the NYC Health Department for the period 2009–2022, which were −46% in PM_2.5_ and −41% in NO_2_ (25).

**Table 1:** Trend analysis for PM_2.5_ and NO_2_: citywide and by borough.

Area	Exposure	Beta estimate^*^	*p*-value	95% CI
New York City	PM_2.5_	−0.35	<0.0001	(−0.41, −0.30)
New York City	NO_2_	−0.59	<0.0001	(−0.68, −0.49)
Bronx	PM_2.5_	−0.4	<0.0001	(−0.47, −0.34)
Bronx	NO_2_	−0.62	<0.0001	(−0.75, −0.49)
Brooklyn	PM_2.5_	−0.35	<0.0001	(−0.41, −0.29)
Brooklyn	NO_2_	−0.64	<0.0001	(−0.74, −0.53)
Manhattan	PM_2.5_	−0.45	<0.0001	(−0.51, −0.39)
Manhattan	NO_2_	−1.08	<0.0001	(−1.19, −0.96)
Queens	PM_2.5_	−0.33	<0.0001	(−0.38, −0.27)
Queens	NO_2_	−0.57	<0.0001	(−0.67, −0.48)
Staten Island	PM_2.5_	−0.34	<0.0001	(−0.40, −0.28)
Staten Island	NO_2_	−0.36	<0.0001	(−0.46, −0.26)

* The regression coefficient estimate is the change in exposure (ppb) per year.

The pollutant decreases were seen in all counties (Appendix Table 2). Over the period 2009–2021, there was a decrease of 39.9% in PM_2.5_ and 32.5% in NO_2_ in the Bronx; in Brooklyn, decreases of 36.8% in PM_2.5_ and 30.8% in NO_2_; in Manhattan, decreases of 41.4% in PM_2.5_ and 41.1% in NO_2_; in Queens, 35.5% decrease in PM_2.5_ and 30.3% in NO_2_; and in Staten Island, 37.3% decrease in PM_2.5_ and 23.7% in NO_2_.

### Residential air modeling of CCCEH cohorts

**Figure 2:**
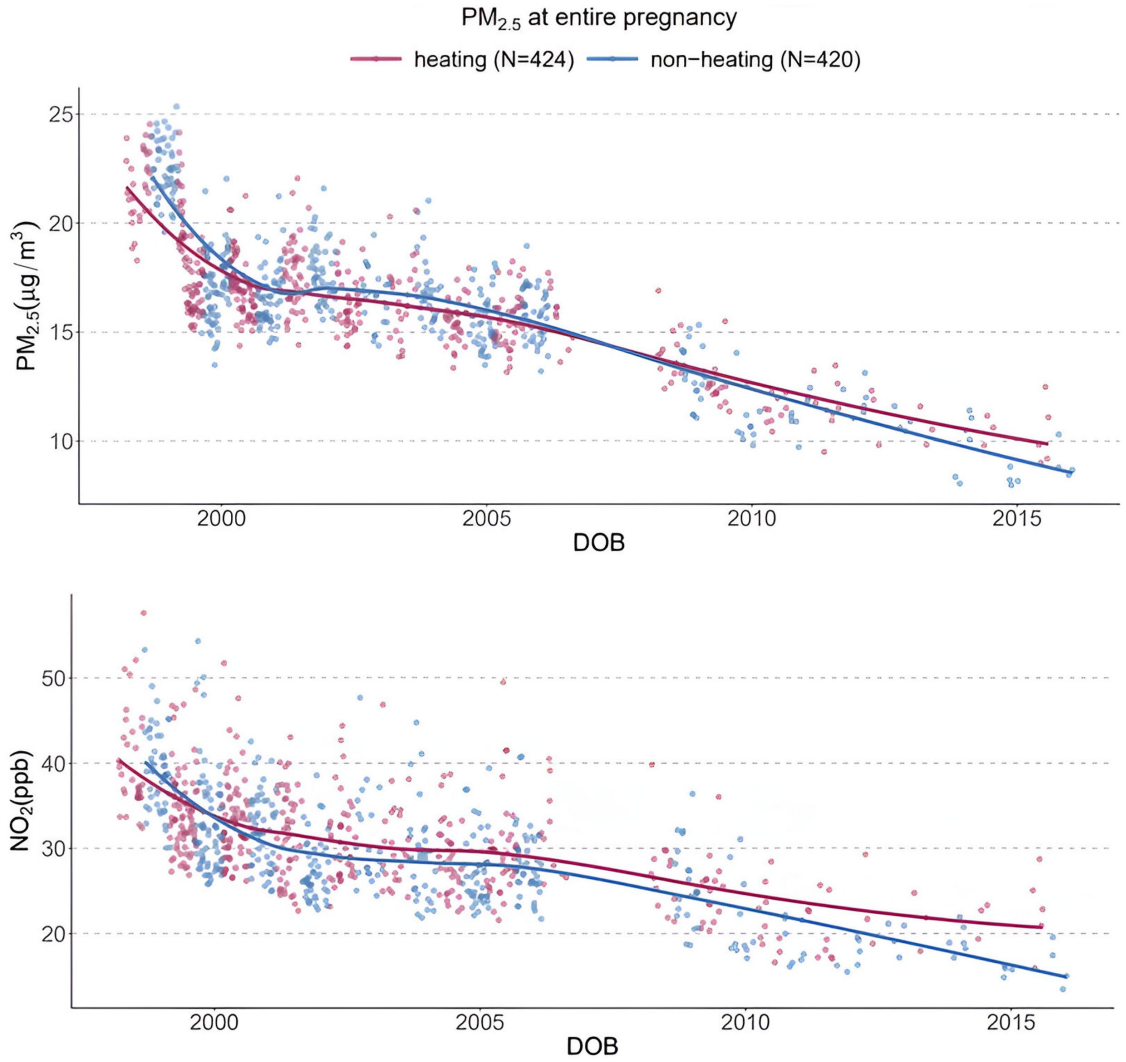
Modeled residential levels of PM_2.5_ and NO_2_ in CCCEH cohorts.

Figure 2 shows the declining residential levels of PM_2.5_ and NO_2_ between 1998 and 2016 in CCCEH cohorts. Means and standard deviations of PM_2.5_ and NO_2_ for each year between 1998 and 2016 are presented in Appendix Table 3, stratified by heating/non-heating season, where the season was designated based on when the mother spent >50% of her pregnancy.

As shown in Table 2, PM_2.5_ decreased by 51.3% overall between 1998 and 2016, with a decrease of 45.7% in the heating- and 55.7% in the non-heating season. NO_2_ decreased by 47.7% overall from the start and the end of the follow-up period, with a decrease of 38.3% in the heating season, and a decrease of 53.5% in the non-heating season.

**Table 2:** Change of PM_2.5_ and NO_2_ based on residential modeling from 1998–1999 to 2014–2016.

		Start 1998–1999^*^	End of the follow-up 2014–2016^*^	Change from start to end of the follow-up period (%)
Heating season	*N*	104	8	
	PM_2.5_ mean (sd)	19.16 (2.60)	10.40 (1.16)	−45.69%
	NO_2_ mean (sd)	36.21 (5.95)	22.36 (3.80)	−38.25%
Non-heating season	*N*	71	14	
	PM_2.5_ mean (sd)	20.78 (2.89)	9.21 (1.20)	−55.69%
	NO_2_ mean (sd)	37.50 (6.11)	17.44 (2.42)	−53.50%
Overall	N	175	22	
	PM_2.5_ mean (sd)	19.82 (2.83)	9.64 (1.30)	−51.34%
	NO_2_ mean (sd)	36.73 (6.03)	19.23 (3.79)	−47.66%

* Data for the first two and last 2 years were averaged to achieve a more stable estimate.

Table 3 shows a significant decrease annually in PM_2.5_ and NO_2_ between 1998 and 2016 from the linear regression models introduced above in both the heating- and non-heating seasons (all with *p* < 0.0001). This was estimated to be a reduction of 0.57 μg/m^3^ in PM_2.5_ per year in the heating season and 0.64 μg/m^3^ per year in the non-heating season. Yearly reductions in NO_2_ were estimated to be 1.00 ppb in the heating season and 1.18 ppb in the non-heating season.

**Table 3:** Results of association analysis between residential air pollutants and time stratified by heating/non-heating season.

	Air pollutants	Beta estimate*	*p*-value	95% CI
Heating season	PM_2.5_	−0.57	<0.0001	(−0.61, −0.53)
	NO_2_	−1.00	<0.0001	(−1.13, −0.87)
Non-heating season	PM_2.5_	−0.64	<0.0001	(−0.67, −0.60)
	NO_2_	−1.18	<0.0001	(−1.30, −1.06)

We also calculated the change in exposure between the start and the end of the follow-up period. To do so, we averaged PM_2.5_/NO_2_ values for the first 2 years (1998–1999) and the last 2 years (2015–2016) to achieve a more stable estimate and computed the change between them.

#### Personal monitoring of PAH in CCCEH cohorts

**Figure 3:**
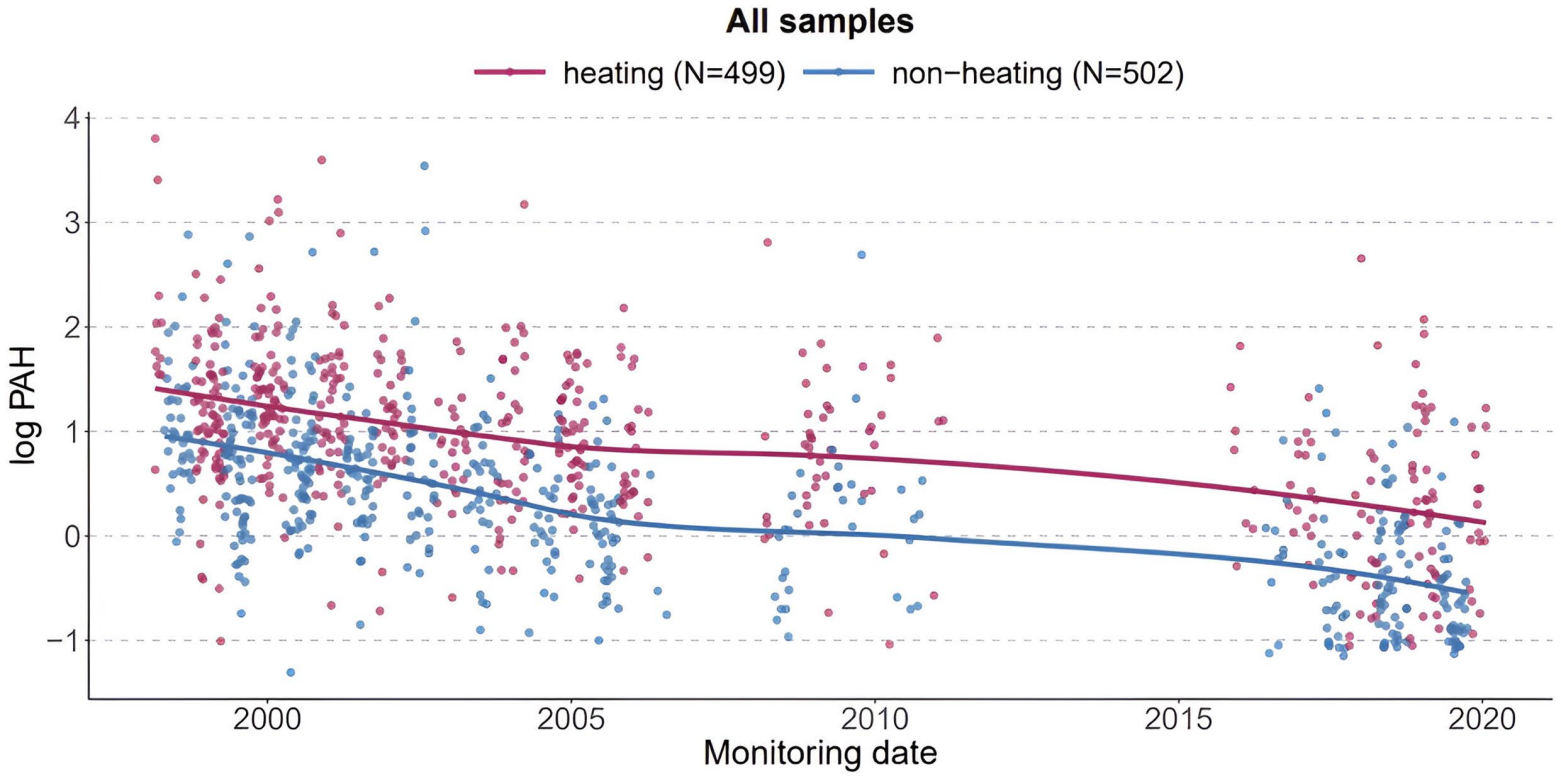
PAH measured by personal air monitors during pregnancy of participants in the CCCEH cohorts.

Figure 3 shows the significant decreasing trend in PAH by whether monitoring took place in the heating or non-heating season, using all samples in the CCCEH cohorts spanning a 20-year period. PAH was estimated to decrease by 0.05 ng/m^3^ each year during the heating season (*p*-value = <0.0001), and 0.06 ng/m^3^ during the non-heating season (p-value = <0.0001) (Table 4). Appendix Table 4 provides detailed yearly data on PAH concentrations, stratified by heating season. As shown in Appendix Table 5, PAH decreased by 62.4% from 1998 to 2020 in the heating season, 78.9% in the non-heating season, and 65.6% overall.

**Table 4:** Results of association analysis between PAH and time (number of years since February 25, 1998) stratified by heating/non-heating season.

	Air pollutants	Beta estimate^*^	*p*-value	95% CI
Heating season	PAH	−0.052	<0.0001	(−0.061, −0.044)
Non-heating season	PAH	−0.064	<0.0001	(−0.071, −0.056)

The beta regression coefficient estimate is change in PAH (ng/m^3^) per year.

* Time unit is the number of years since February 25, 1998.

#### Summary of results

Across all three settings, the reductions in levels of air pollution were significant. Comparing the estimated yearly decreases for PM_2.5_ and NO_2_ for the citywide and the residential cohort data, we found somewhat higher reductions in the cohort data compared to the citywide data. Citywide, there was an estimated decrease of 0.35 μg/m^3^ per year in average PM_2.5_, and a decrease of 0.59 ppb per year in average NO_2_. In the residences of our CCCEH cohorts, average annual PM_2.5_ decreased by 0.57 μg/m^3^ per year and NO_2_ decreased by 1.00 ppb per year in the heating season; in the non-heating season, PM_2.5_ decreased by 0.64 μg/m^3^ per year and NO_2_ decreased by 1.18 ppb per year. Additionally, PAH decreased by 0.05 ng/m^3^ and 0.06 ng/m^3^ per year for the heating and non-heating seasons, respectively, and 65.6% overall.

## Discussion

Between 1998 and 2021, there were significant downward trends in air pollution measured at the level of the city and within CCCEH birth cohorts carried out in northern Manhattan and the South Bronx. The trends are consistent with a cumulative effect of multiple policies enacted over the study period. The citywide percent declines of PM_2.5_ and NO_2_ annually were lower than those in the cohorts. The largest overall decline over the 12-year period 1998–2020 was seen in PAH levels based on personal air monitoring: 62.4% reduction in the heating season and 78.9% in the non-heating season. Higher overall pollution levels were expected in the heating season due to increased usage of boilers, a common heating system in NYC buildings, as well as increased fossil fuel usage from other heat sources and other devices reflecting increased time spent indoors. Contrary to expectation, the mean PM_2.5_ level at residences of cohort women in the heating season in the early years was somewhat lower than the mean level during the non-heating season. This is likely because in early years for 1998–2000, there were fewer samples collected during the heating season, and there were two outliers in the non-heating season group.

The study has a number of limitations. We were unable to ascribe changes in air quality to any single policy because the successive policies overlapped in terms of operation and targeted the same sources. We can only infer that they had a cumulative effect on air quality in the city. A further limitation of this study is the lack of inclusion of all minor rules and regulations over the time period analyzed. While the methodology used by Rhodes-Bratton categorized all legislation, including minor laws, over their chosen time period, we used an approach that focused solely on “major” policies, defined as legislation that has been reported by ourselves and others to have had a measurable impact on air quality. Additionally, we entered the date of enactment of the original legislation because the dates of actual implementation for all policies were not consistently available. As explained above, we also did not include statewide or national policies, which undoubtedly impacted air quality during the study period.

We were unable to adequately assess the impacts on disparities in exposure at the neighborhood level. However, the citywide data show benefits in air quality across all five boroughs, including the Bronx, which has had historically higher levels of air pollution and elevated rates of associated adverse health effects. Benefits were also seen in Manhattan overall, where the communities in Northern Manhattan—Harlem and Washington Heights—also have a history of elevated exposure to air pollution. Exploratory analysis of whether boroughs with a lower percentage of white people and higher percentage of people in poverty suggested that the Bronx, which has the highest percentage of people in poverty and the lowest percentage of White population, saw the second-greatest decrease in PM_2.5_, and the second-greatest decrease in NO_2_ among the five boroughs. The greatest PM_2.5_ decrease was seen in Staten Island, a borough which is 60% White; and the greatest NO_2_ decrease was seen in Manhattan, which is 47% White; these two boroughs have the highest percentages of White populations in NYC, and both have lower percentages of people in poverty compared to the other boroughs (Appendix Table 2). These findings are mixed compared with other reports on the generally inequitable distribution of air pollution in terms of race, ethnicity, and income (10, 45). However, in our cohorts drawn from the predominantly lower-income communities of color in Northern Manhattan and South Bronx, we see substantial improvement. Nonetheless, the higher baseline pollution levels and incidence rates, and lower access to resources in these communities point to a major inequality issue that requires further environmental, health, and social policy interventions (46). We note that direct extrapolation of our results to other populations is difficult; however, large cities across the country have similar disparities in exposure, making our findings more broadly relevant.

Analysis of trends in the rates of illnesses and developmental disorders that have been associated with the three pollutants analyzed were outside of the scope of this analysis and will be the subject of future research. However, we note that our prior analysis of the air quality improvement in NYC during the COVID-19 shutdown period of March 15 to May 15, 2020 resulted in a sharp (23%) reduction of PM_2.5_ in New York City. Modeled extrapolations from these lowered pollution levels, simulating a scenario where these reduced levels of pollution were sustained over the following five years, estimated thousands of avoided cases of illness and death including in children, with associated economic benefits ranging from $31.8 to $77 billion (47). Given the 37% reduction in citywide PM_2.5_ concentrations reported here, the health benefits due to clean air policies would likely be even greater.

## Conclusion

While it is not possible to link improved air quality to a single policy, our analysis provides evidence of a cumulative beneficial effect of clean air and climate policies enacted between 1998 and 2021 both city-wide and in our cohorts residing in communities that have been disproportionately affected by air pollution. Because of the known significant associations between the pollutants studied and multiple adverse health effects, there are important implications for health benefits, particularly for children, who are especially vulnerable to these exposures. The results support further environmental and social policy changes to prevent the serious health impacts of air pollution from fossil fuel emissions.

## Data Availability

The original contributions presented in the study are included in the article/Supplementary material, further inquiries can be directed to the corresponding author.

## Footnotes

^1^ https://a816-dohbesp.nyc.gov/IndicatorPublic/data-features/nyccas/
