# Supplementary material for: Major air pollution and climate policies in NYC and trends in NYC air quality 1998–2021
Source: Front Public Health. 2024 Oct 16;12:1474534. doi: 10.3389/fpubh.2024.1474534 (PMC11521894; doi:10.3389/fpubh.2024.1474534)
Supplement: Supplementary file 1 [file Table_1.docx]

**Appendix**

**Table 1. Major air pollution emission reduction policies enacted during the period 1998 – 2021**

| **Targeted Emissions Source** | **Name** | **Year enacted** | **Year(s) Implemented** | **Description** | **Impact Report** |
| --- | --- | --- | --- | --- | --- |
| Metropolitan Transportation Authority (MTA) bus fleet | Clean Fuel Bus Program | 2000 | 2000 - 2004 | Switched all diesel buses in the fleet to ultra-low sulfur fuel  Replaced two-stroke engines with new four-stroke engines  Retrofitted diesel buses with catalytic diesel particulate filters  Incorporated more hybrid electric and Compressed Natural Gas buses in fleet composition | Lovasi et al., 2022 |
| Medallion taxi fleet (“yellow cabs”) | Clean Air Taxi: Local Law 2005/072 | 2005 | 2005 | Mandated Taxi and Limousine Commission (TLC) approval of one or more hybrid vehicle models for use as taxicabs | Fry et al., 2020 |
| Medallion taxi fleet (“yellow cabs”) | Clean Air Taxi: Local Law 2006/018 | 2006 | 2006 | Mandated that at least 9% of new medallions sold be restricted to hybrid or compressed natural gas vehicles | Fry et al., 2020 |
| Medallion taxi fleet (“yellow cabs”) | Clean Air Taxi: Local Law 2006/052 | 2006 | 2006 | Incentivized the purchase of low-emission taxicabs by extending the useful lifetime of taxi models that are classified as “clean air” vehicles by the United States Environmental Protection Agency (EPA) | Fry et al., 2020 |
| Residential and commercial boilers for heat and hot water | Clean Heat Program: Local Law 2010/043 | 2010 | 2012 | Mandated conversion from No. 6 oil in boilers to cleaner burning fuel sources, including lower sulfur No. 2 fuel, biodiesel or natural gas, and No. 4, a blend of No. 2 and No. 6 oils  Mandated sulfur content in heating oil No. 4 to be reduced from 3,000 ppm to 1,500 ppm  Mandated all heating oil must contain 2% biofuel | Zhang et al., 2021 |
| Commercial char broilers | Local Law 2015/038 | 2015 | 2016 | Required emission control devices to be installed on all commercial char broilers in restaurants | Shukla et al., 2022 |
| Buildings >25,000 ft2 (2,323 m2) | Climate Mobilization Act: Local Law 2019/097 | 2019 | 2019 | Required all mid-size and large buildings to reduce the GHG emissions associated with their energy use from the 2005 baseline; 40% by 2030, and 80% by 2050 | Salimifard et al., 2022 |

**Table 2. Income, race, and pollutant decrease percentages by NYC borough**

| **Borough** | **% People in Poverty (2018)** | **% White Population**  **(2018)** | **PM_2.5_ decrease 2009-2021 (µg/m^3^)** | **% PM_2.5_ decrease 2009-2021** | **NO_2_ decrease 2009-2021 (ppb)** | **% NO_2_ decrease 2009-2021** |
| --- | --- | --- | --- | --- | --- | --- |
| All boroughs | 17.3% | 31.9% | 3.89 | 37.3% | 7.04 | 31.0% |
| Bronx | 27.4% | 8.9% | 4.41 | 39.9% | 7.98 | 32.5% |
| Brooklyn | 19.0% | 36.2% | 3.87 | 36.8% | 7.43 | 30.8% |
| Manhattan | 15.5% | 46.8% | 5.22 | 41.4% | 13.92 | 41.1% |
| Queens | 11.5% | 24.7% | 3.57 | 35.5% | 6.73 | 30.3% |
| Staten Island | 11.4% | 60.1% | 3.66 | 37.3% | 4.00 | 23.7% |

**Table 3. Summary data of residential PM_2.5_ and NO_2_ in CCCEH cohorts stratified by heating/non-heating season**

| **DOB Year** | **Heating season (N=424)** | | | **Non-heating season (N=420)** | | |
| --- | --- | --- | --- | --- | --- | --- |
|  | N | PM_2.5_ (µg/m^3^)  Mean (sd) | NO_2_ (ppb)  Mean (sd) | N | PM_2.5_ (µg/m^3^)  Mean (sd) | NO_2_ (ppb)  Mean (sd) |
| 1998 | 31 | 21.76 (1.64) | 40.44 (6.20) | 10 | 22.68 (1.38) | 40.63 (6.18) |
| 1999 | 73 | 18.05 (2.09) | 34.42 (4.87) | 61 | 20.47 (2.96) | 36.98 (5.99) |
| 2000 | 68 | 17.08 (1.36) | 33.28 (4.98) | 62 | 16.89 (1.53) | 32.62 (5.12) |
| 2001 | 46 | 17.66 (1.60) | 31.50 (5.27) | 56 | 17.25 (1.51) | 30.55 (4.43) |
| 2002 | 39 | 16.28 (1.21) | 30.98 (4.85) | 37 | 17.28 (1.25) | 28.91 (5.26) |
| 2003 | 25 | 16.64 (1.77) | 30.58 (5.23) | 21 | 16.58 (1.58) | 29.58 (4.37) |
| 2004 | 33 | 15.57 (1.00) | 28.81 (4.37) | 33 | 16.49 (1.37) | 29.01 (5.43) |
| 2005 | 37 | 15.55 (1.26) | 29.64 (5.60) | 50 | 15.74 (1.27) | 27.77 (4.57) |
| 2006 | 10 | 15.84 (1.34) | 31.86 (5.24) | 26 | 15.46 (1.10) | 27.48 (3.06) |
| 2008 | 13 | 14.01 (1.22) | 27.23 (4.73) | 6 | 13.63 (0.58) | 26.50 (4.07) |
| 2009 | 16 | 12.54 (0.93) | 25.07 (3.91) | 17 | 12.87 (1.48) | 25.18 (5.16) |
| 2010 | 10 | 11.43 (0.60) | 21.42 (3.79) | 12 | 10.86 (0.73) | 21.06 (4.00) |
| 2011 | 9 | 11.75 (1.23) | 20.56 (3.51) | 6 | 11.56 (1.05) | 17.50 (2.07) |
| 2012 | 3 | 11.35 (1.34) | 23.26 (5.41) | 6 | 11.79 (0.73) | 18.28 (2.12) |
| 2013 | 3 | 10.65 (0.89) | 21.51 (3.42) | 3 | 10.67 (0.22) | 19.08 (1.87) |
| 2014 | 3 | 10.53 (0.68) | 21.80 (2.13) | 6 | 9.91 (1.40) | 19.32 (1.73) |
| 2015 | 5 | 10.33 (1.46) | 22.70 (4.77) | 6 | 8.72 (0.85) | 16.62 (1.67) |
| 2016 | NA | NA | NA | 2 | 8.56 (0.16) | 14.23 (1.09) |

**Table 4. Annual average PAH concentrations in the CCCEH cohorts, stratified by heating/non-heating season**

| Year | **Heating season (N=499)** | | **Non-heating season (N=502)** | |
| --- | --- | --- | --- | --- |
|  | N | PAH mean (sd) (ng/m^3^) | N | PAH mean (sd) (ng/m^3^) |
| 1998 | 11 | 11.63 (13.36) | 37 | 3.53 (3.07) |
| 1999 | 69 | 3.64 (2.39) | 68 | 2.76 (2.72) |
| 2000 | 64 | 4.93 (4.50) | 64 | 2.63 (2.23) |
| 2001 | 47 | 4.65 (5.56) | 44 | 2.22 (2.14) |
| 2002 | 34 | 3.61 (2.07) | 33 | 3.56 (6.40) |
| 2003 | 24 | 2.58 (1.34) | 30 | 1.71 (0.91) |
| 2004 | 34 | 3.56 (4.12) | 26 | 1.38 (0.72) |
| 2005 | 45 | 2.79 (1.37) | 40 | 1.27 (0.77) |
| 2006 | 26 | 2.68 (1.98) | 3 | 0.95 (0.73) |
| 2008 | 6 | 3.91 (6.23) | 14 | 0.86 (0.43) |
| 2009 | 24 | 2.67 (1.46) | 10 | 3.14 (4.14) |
| 2010 | 11 | 2.74 (1.64) | 8 | 1.02 (0.47) |
| 2011 | 4 | 3.31 (2.51) | 0 | NA |
| 2016 | 8 | 2.48 (1.86) | 10 | 0.99 (0.62) |
| 2017 | 17 | 1.68 (0.83) | 26 | 0.96 (0.98) |
| 2018 | 22 | 1.77 (3.05) | 50 | 0.85 (0.55) |
| 2019 | 38 | 1.91 (1.76) | 39 | 0.64 (0.48) |
| 2020 | 15 | 1.44 (0.96) | 0 | NA |
| All | 499 | 3.46 (3.94) | 502 | 1.99 (2.61) |

* We omit the years with less than 5 samples.

**Table 5. Change of PAH from start (1998-1999) to end (2019-2020) of follow-up period of the CCCEH cohorts**

|  | | **Start**  **1998-1999** | **End**  **2019-2020** | **Change from**  **Start to End (%)** |
| --- | --- | --- | --- | --- |
| **Heating season** | N | 80 | 53 |  |
|  | PAH mean (sd) | 4.73 (5.93) | 1.78 (1.58) | -62.42% |
| **Non-heating season** | N | 105 | 39 |  |
|  | PAH mean (sd) | 3.03 (2.86) | 0.64 (0.48) | -78.85% |
| **Overall** | N | 185 | 92 |  |
|  | PAH mean (sd) | 3.77 (4.52) | 1.30 (1.36) | -65.59% |

*Data for the first two and last two years were averaged to achieve an adequate sample size.
